# Synthesis of a hexasaccharide partial sequence of hyaluronan for click chemistry and more

**DOI:** 10.3762/bjoc.11.67

**Published:** 2015-04-30

**Authors:** Marina Bantzi, Stephan Rigol, Athanassios Giannis

**Affiliations:** 1 Universität Leipzig, Institut für Organische Chemie, Johannisallee 29, 04103 Leipzig, Germany; 2 Current address Rice University, Department of Chemistry, BioScience Research Collaborative, 6100 Main Street, Houston, Texas 77005, USA

**Keywords:** azide, click chemistry, extracellular matrix, hexasaccharide, hyaluronan, multivalency

## Abstract

In the present work, the synthesis of a hexasaccharide partial sequence of hyaluronan equipped with a terminal azido moiety is reported. This hexasaccharide can be used for the attachment on surfaces by means of click chemistry and after suitable deprotection for biophysical studies.

## Introduction

Much effort has been exerted during the last years to refine current knowledge about the biology of the extracellular matrix (ECM) [1]. While in the past, it was only regarded as a “space filler” among the cells, nowadays it is well known that the ECM composes the ideal microenvironment for cells in order to interact with each other and also for supporting signaling between ECM macromolecules and intracellular components [2]. Besides water, the ECM consists of electrolytes, amino acids, monosaccharides, fibrous proteins (collagens), glycosaminoglycans (GAGs), and proteoglycans (PGs). The latter are complex proteins containing at least one covalently bound glycosaminoglycan part [3]. GAGs are long, unbranched polysaccharides comprising repeating disaccharide units, which are constituted of a hexosamine and an uronic acid. These repeating disaccharide units are used for the classification of GAGs [4].

Hyaluronic acid, a member of the GAG family, owes its name to the Greek word “ΰαλος” (= glass) since it was first isolated in 1934 from the vitreous body of the bovine eye [5]. Its structure was elucidated in 1954 [6] and since 1986 it is known as “hyaluronan” (HA) [7]. HA is an unbranched polysaccharide, whose disaccharide repeating unit consists of *N*-acetylglucosamine and D-glucuronic acid conjoined through β-(1→3) and β-(1→4)-glycosidic bonds. Hyaluronan has an average size of 15–20 kDa and does not form PGs, in contrast to the other GAGs, which are synthesized in the Golgi apparatus or the endoplasmic reticulum [8]. HA is enzymatically produced by three glycosyltransferases (HA synthases: HAS 1, 2 and 3) in the cellular plasma membrane and its chain can reach a mass of 10^2^–10^4^ kDa [9]. Despite its simple structure HA can trigger many signaling pathways depending on its fragments' size, thus representing an interesting target in pharmacotherapy. It is involved in tissue repair and wound healing; it serves as space filler, lubricant, protector of the joints and water storage [4]. In addition, HA is able to interact with three major classes of cell surface receptors, namely CD44 (cluster of differentiation 44), RHAMM (receptor for HA-mediated motility) and ICAM-1 (intracellular adhesion molecule-1) [10–11]. CD44 is a heterogeneous, transmembrane glycoprotein which is overexpressed on the surface of cancer stem cells [11–12] and plays a crucial role in the development of different types of cancer [13]. It seems that short fragments of HA (3–25 disaccharides) cause a pro-angiogenic effect in contrast to longer ones depending on the activity of this receptor [9]. Hence, well-defined oligomers related to HA are highly desired as novel pharmacotherapy targets.

Syntheses of HA disaccharides appear for the first time in literature in 1962 from Jeanloz et al. [14] and Takanashi et al. [15]. Since then, many efforts have been done in this field resulting in the synthesis of longer HA fragments which were bearing either a free reducing end or a non-functionalized aglycone [16–18]. In 2007, a study focusing on the synthesis of HA sequences which could be functionalized and used for biological studies yielded oligosaccharides bearing an alkyl-azide [19]. Besides the results of this work as well as that from Hsieh-Wilson et al. [20] and van der Marel et al. [21] both published *O*-1-allyl-equipped HA subunits, we reported recently the first synthesis of a ^13^C-labeled HA tetramer for ongoing biophysical studies [22]. Different methodologies were used to establish the glycosidic linkages; most important was the reaction's stereochemical outcome. Elongation of the synthesized oligosaccharides was easily done, since the TBS-protection is selectively cleavable. The anomeric allyl moiety permits varieties of feasible modifications including the introduction of an azido group. In the frame of a research project aiming the investigation of protein–GAG binding a convergent synthesis of a HA hexamer with a suitably modified aglycone is described herein.

## Results and Discussion

**Scheme 1:**
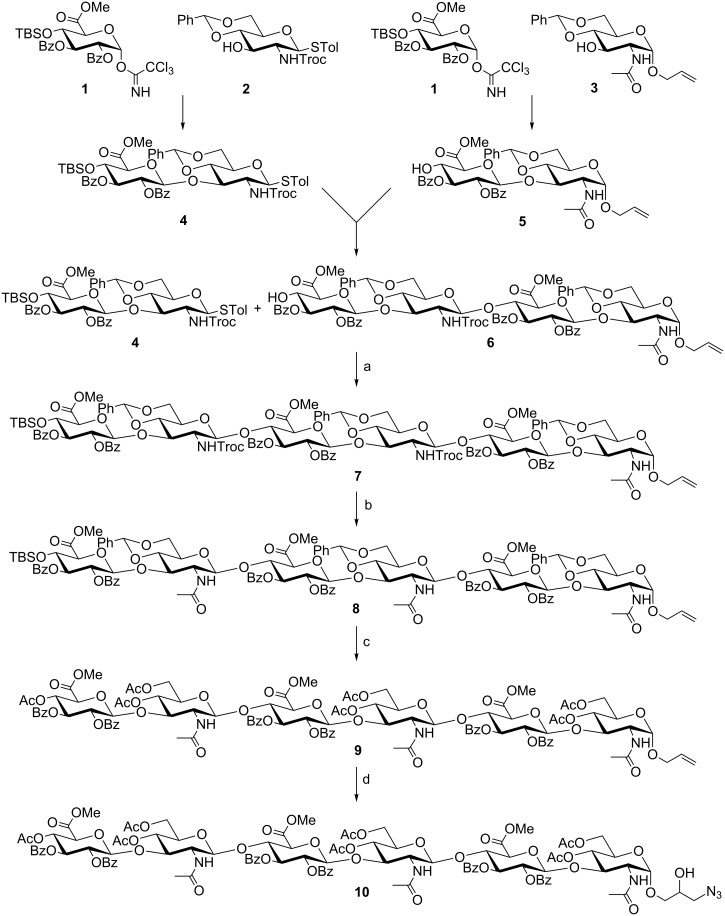
Synthesis of hexasaccharide **10**. Conditions: a) TfOH, NIS, 4 Å molecular sieves, DCM, 0 °C to rt; b) 1. Zn, AcOH; 2. Ac_2_O, pyridine, 50% (over 2 steps); c) 1. HF·pyridine; 2. Ac_2_O, pyridine, 70%; d) 1. DMDO, acetone, −78 °C to rt; 2. NaN_3_, DMF, 70%.

The synthetic cascade to the desirable hexasaccharide **10** is presented in Scheme 1. Trichloroacetimidate **1** [20,23] was linked with glycosyl acceptor **2** [24–25] using TMSOTf as promoter to obtain disaccharide **4** in 90% yield. Likewise, reaction of glycosyl donor **1** with monosaccharide **3** [26] and subsequent *O*-TBS group cleavage with Olah's reagent [27], afforded disaccharide **5** in 86% yield. Thence, both disaccharides were coupled through initial activation of **4** with NIS and TfOH to furnish the corresponding protected tetrasaccharide. Furthermore, treatment of the glycosylation product with Olah's reagent and an additional amount of pyridine generated the tetrasaccharide glycosyl acceptor **6** by removal of the TBS group at O-4''' in 59% yield [22]. The excess amount of pyridine is necessary in order to avoid cleavage of the benzylidene acetals. Following the same concept, fully protected hexasaccharide **7** was synthesized. Therefore, thioglycoside **4** was activated with NIS and TfOH and subsequently combined with tetrasaccharide **6**. The underlying protecting group pattern with a selectively cleavable silyl group at the non-reducing end of the saccharide sequence permits the further elongation by additional iterative cycles based on the presented methodology. Then, the *N*-Troc groups were cleaved under mild reducing conditions (Zn, AcOH) [28] and subsequently the liberated amino groups were acetylated to furnish compound **8**. Eventually, the silyl group and all benzylidene moieties were removed by treatment with Olah's reagent to give, after acetylation, derivative **9**. Finally, oxidation of the terminal olefinic double bond with Murray's reagent [29–30] yielded the analogous epoxide that was treated with NaN_3_ in order to afford the desired azido-modified hexasaccharide **10**.

## Conclusion

In conclusion, hexasaccharide **10** was successfully prepared in 26 steps and is readily equipped with a terminal azido group. Thus, allowing it to be used for surface modification via click chemistry. After suitable deprotection it can be used for biophysical studies by interaction with an alkyne group of suitably prepared proteins or proteoglycans giving the opportunity to gain deeper insights into ECM processes. Eventually, this knowledge can be employed during the development of artificial extracellular matrices for basic research in the field of wound healing in skin and bone injuries.

## Supporting Information

File 1 Experimental details, analytical data and copies of ^1^H and ^13^C NMR spectra for the newly synthesized compounds.
