# Supplementary material for: Synthesis of a hexasaccharide partial sequence of hyaluronan for click chemistry and more
Source: Beilstein J Org Chem. 2015 Apr 30;11:604–7. doi: 10.3762/bjoc.11.67 (PMC4462852; doi:10.3762/bjoc.11.67)
Supplement: File 1 — Experimental details, analytical data and copies of 1H and 13C NMR spectra for the newly synthesized compounds. [file Beilstein_J_Org_Chem-11-604-s001.pdf]

Supporting Information
for
Synthesis of a hexasaccharide partial sequence of hyaluronan for click chemistry and more

Marina Bantzi[1], Stephan Rigol[1,2], and Athanassios Giannis*[1]

Address: [1]Universität Leipzig, Institut für Organische Chemie, Johannisallee 29, 04103 Leipzig, Germany and [2]Current address Rice University, Department of Chemistry, BioScience Research Collaborative, 6100 Main Street, Houston, Texas 77005, USA

Email: Athanassios Giannis - giannis@uni-leipzig.de

* Corresponding author

Experimental details, analytical data and copies of ^{1}H and ^{13}C NMR spectra for the newly synthesized compounds

Table of contents

1. General experimental methods S2
2. Synthesis overview S3
3.1 Prop-2-en-1-yl 2,3-di-*O*-benzoyl-4-*O*-[*tert*-butyl(dimethyl)silyl]-6-methyl-D-glucopyranuronosyl-(1→3)-2-(acetylamino)-4,6-*O*-benzylidene-2-deoxy-β-D-glucopyranosyl-(1→4)-2,3-di-*O*-benzoyl-6-methyl-β-D-glucopyranuronosyl-(1→3)-2-(acetylamino)-4,6-*O*-benzylidene-2-deoxy-β-D-glucopyranosyl-(1→4)-2,3-di-*O*-benzoyl-6-methyl-β-D-glucopyranuronosyl-(1→3)-2-(acetylamino)-4,6-*O*-benzylidene-2-deoxy-α-D-glucopyranoside (**8**) S4
3.2 Prop-2-en-1-yl 4-*O*-acetyl-2,3-di-*O*-benzoyl-6-methyl-D-glucopyranuronosyl-(1→3)-4,6-di-*O*-acetyl-2-(acetylamino)-2-deoxy-β-D-glucopyranosyl-(1→4)-2,3-di-*O*-benzoyl-6-methyl-β-D-glucopyranuronosyl-(1→3)-4,6-di-*O*-acetyl-2-(acetylamino)-2-deoxy-β-D-glucopyranosyl-(1→4)-2,3-di-*O*-benzoyl-6-methyl-β-D-glucopyranuronosyl-(1→3)-4,6-di-*O*-acetyl-2-(acetylamino)-2-deoxy-α-D-glucopyranoside (**9**) S5
3.3 3-Azido-2-hydroxypropyl 4-*O*-acetyl-2,3-di-*O*-benzoyl-6-methyl-D-glucopyranuronosyl-(1→3)-4,6-di-*O*-acetyl-2-(acetylamino)-2-deoxy-β-D-glucopyranosyl-(1→4)-2,3-di-*O*-benzoyl-6-methyl-β-D-glucopyranuronosyl-(1→3)-4,6-di-*O*-acetyl-2-(acetylamino)-2-deoxy-β-D-glucopyranosyl-(1→4)-2,3-di-*O*-benzoyl-6-methyl-β-D-glucopyranuronosyl-(1→3)-4,6-di-*O*-acetyl-2-(acetylamino)-2-deoxy-α-D-glucopyranoside (**10**) S7
4. NMR spectra S9
5. References S14

1. General experimental methods

All reactions were carried out in flame-dried flasks under an argon atmosphere. Dry dichloromethane was freshly distilled from SICAPENT (phosphorus pentoxide on solid support with indicator) and dry tetrahydrofuran (THF) was freshly distilled from potassium. Dry *N*,*N*-dimethylformamide, dry methanol, dry acetonitrile, dry pyridine as well as all reagents were purchased from ABCR, Acros Organics, Carbolution, Fluka, Fluorochem, Merck, Sigma–Aldrich and VWR International and used without further purification. All reactions were performed at room temperature (22–25 °C) and ambient pressure (1013.25 hPa) unless otherwise indicated. Yields refer to chromatographically and spectroscopically pure compounds.

Analytical TLC was carried out on alumina plates precoated with silica gel (Merck 60-F_{254}, 0.2 mm). The visualization was accomplished under UV light (254 nm) and through coloring with Seebach [10.0 g $Ce(SO_4)_2 \cdot H_2O$, 25.0 g molybdophosphoric acid hydrate, 60.0 mL H_2SO_4 concentrated and 940 mL H_2O; the solution was diluted 1:1 with H_2O and the development was achieved by a stream of hot air (350 °C)] or ninhydrin [0.30 g ninhydrin, 3.0 mL acetic acid and 97 mL ethanol, the detection was achieved with stream of hot air (350 °C)] staining solution. Flash column chromatography was performed on silica gel 60 (40–63 µm) from Sigma–Aldrich using a forced flow of eluent. Concentration under reduced pressure carried out by rotary evaporation at 40 °C at the appropriate pressure.

NMR spectra were recorded on: Varian “Mercury plus-300” (300 MHz: ^{1}H NMR, 75 MHz: ^{13}C NMR), “Mercury-plus 400” (400 MHz: ^{1}H NMR, 100 MHz: ^{13}C NMR) and “Bruker Avance-700” (700 MHz: ^{1}H NMR, 175 MHz: ^{13}C NMR). The residual solvent signal is used as internal standard (chloroform-d_1: 7.26 (^{1}H NMR), 77.16 (^{13}C NMR); methanol-d_4: 3.31 (^{1}H NMR), 49.00 (^{13}C NMR); dimethyl sulfoxide-d_6: 2.50 (^{1}H NMR), 39.52 (^{13}C NMR). The chemical shifts δ are reported in ppm and multiplicities are classified using the following abbreviations: singlet (s), doublet (d), triplet (t), quartet (q), multiplet (m) and combinations thereof. Coupling constants, *J* are given in Hertz (Hz), followed by the integrated proton number and signal interpretation. High resolution ESI mass spectra were obtained on a Bruker Daltonics FT-ICR-MS “APEX II” [7 T] mass spectrometer. IR spectra were recorded on an FTIR Genesis ATI spectrometer from Mattson/Unicam and FT/IR-4100 type A spectrometer from Jasco. The compounds were measured as KBr pellet. Optical rotation data were obtained using a fully automatic polarimeter Polartronic MHz-8 (Schmidt & Haensch) at the sodium-D line (λ = 598.59 nm). Melting points were measured on a Melting Point B-540 melting point apparatus from Büchi and are uncorrected.

Monosaccharides **1**–**3** were synthesized according to previously published syntheses [1-5], whereas synthesis of compounds **4**–**6** was based on earlier work of our group [6].

2. Synthesis overview

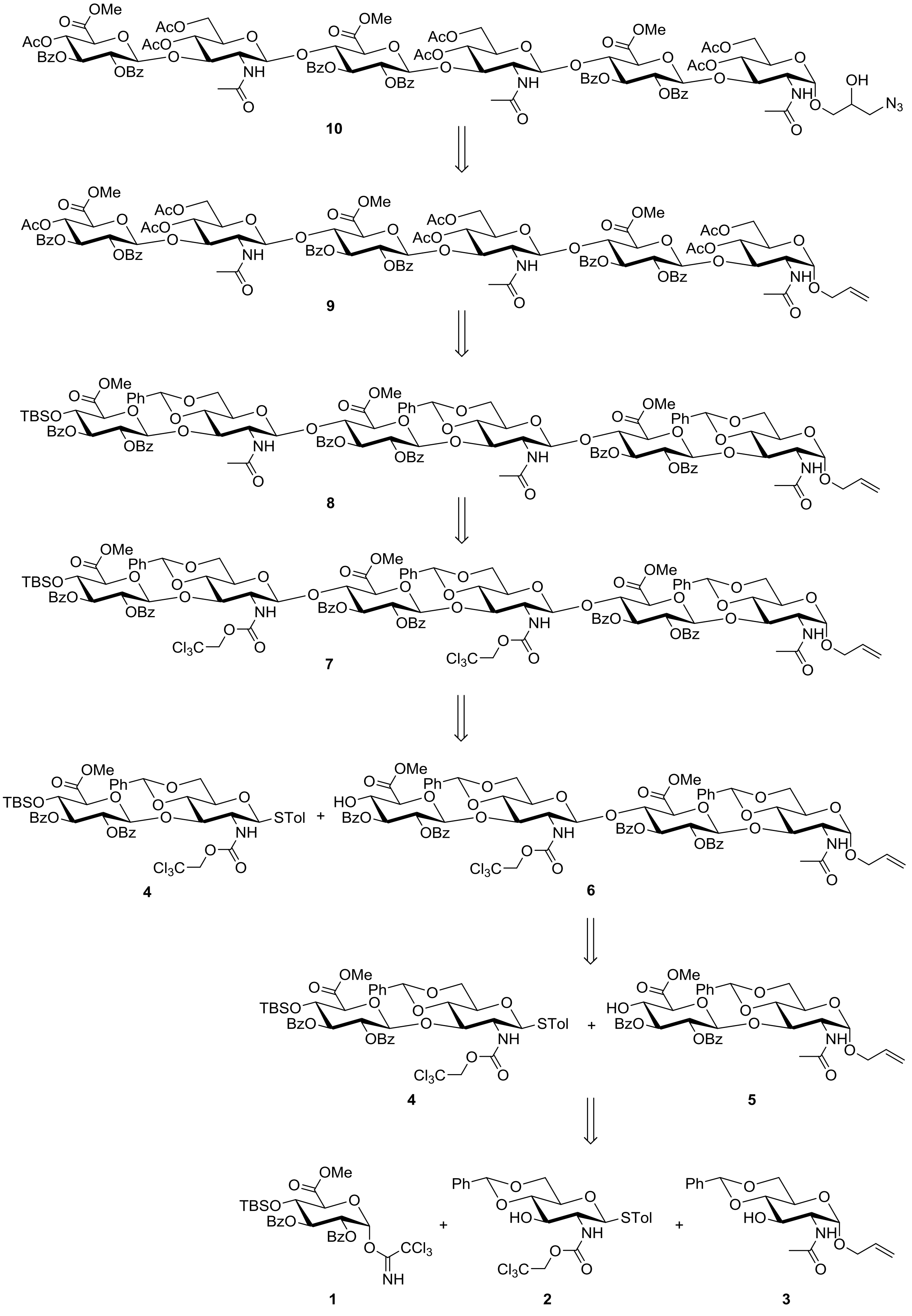

3.1 Prop-2-en-1-yl 2,3-di-*O*-benzoyl-4-*O*-[*tert*-butyl(dimethyl)silyl]-6-methyl-D-glucopyranuronosyl-(1→3)-2-(acetylamino)-4,6-*O*-benzylidene-2-deoxy-β-D-glucopyranosyl-(1→4)-2,3-di-*O*-benzoyl-6-methyl-β-D-glucopyranuronosyl-(1→3)-2-(acetylamino)-4,6-*O*-benzylidene-2-deoxy-β-D-glucopyranosyl-(1→4)-2,3-di-*O*-benzoyl-6-methyl-β-D-glucopyranuronosyl-(1→3)-2-(acetylamino)-4,6-*O*-benzylidene-2-deoxy-α-D-glucopyranoside (8)

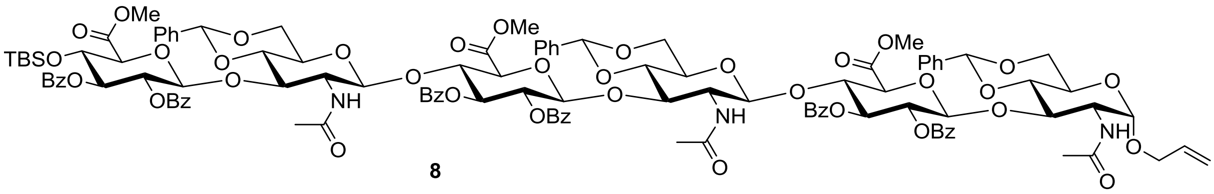

Thioglycoside **4** (475 mg, 447 µmol, 1.5 equiv) and alcohol 6 (467 mg, 298 µmol, 1.0 equiv) were coevaporated with toluene (3 × 3 mL) and dried under high vacuum for 3 h. The mixture was dissolved in dichloromethane (45 mL) and transferred through an injection needle into a flask containing activated 4 Å molecular sieves (1.7 g). Then, the mixture was stirred for 1 h at room temperature. Afterwards, the suspension was cooled to 0 °C and *N*-iodosuccinimide (107 mg, 476 µmol, 1.6 equiv) and trifluoromethanesulfonic acid (10.5 µL, 18.3 mg, 119 µmol, 0.4 equiv) were added and the reaction mixture was stirred for 11 h at room temperature. The reaction was quenched with triethylamine (36 µL) and all solids were filtered off. All volatiles were removed under reduced pressure and the residue was passed through a short column [R_f: 0.44 (*n*-hexane/ethyl acetate = 4:6 v/v)] to yield crude product **7** (~ 600 mg) as a white solid that was used in the next reaction without further purification.

To a solution of the obtained product **7** (600 mg, 239 µmol, 1.0 equiv) in acetic acid (15 mL) activated zinc (2.0 g) was added and the reaction mixture was left to stir at room temperature for 16 h. Then, all solids were filtered off and all volatiles were removed under reduced pressure. The remaining solid residue was dried under high vacuum for 6 h. Afterwards it was taken up in pyridine (20 mL) and acetic anhydride (10 mL) was added dropwise. The reaction mixture was stirred at room temperature for 15 h. Then, the solution was diluted with dichloromethane (150 mL) and carefully quenched by addition of ice cooled saturated aqueous sodium hydrogen carbonate solution (200 mL). The organic phase was washed with saturated aqueous cupric sulfate solution (5 × 50 mL), dried over Na_2SO_4 and the solvents were removed under reduced pressure. The crude product was purified by column chromatography (*n*-hexane/ethyl acetate, 4:6 → 1:9 v/v) to afford hexasaccharide **8** (335 mg, 150 µmol, 50%) as a white solid.

R_f: 0.12 (*n*-hexane/ethyl acetate = 4:6 *v/v*).

^{1}H NMR: (400 MHz, $CDCl_3$) δ [ppm] −0.26 (s, 3 H, -Si-C**H$_3$**), −0.12 (s, 3 H, -Si-C**H$_3$**), 0.67 (s, 9 H, -Si-C(C**H$_3$**)$_3$), 1.35 (s, 3 H, -CO-C**H$_3$**), 1.37 (s, 3 H, -CO-C**H$_3$**), 1.68 (s, 3 H, -CO-C**H$_3$**), 1.81-1.88 (m, 2 H), 1.95 (s, 1 H), 2.07 (s, 1 H), 2.44-2.53 (m, 2 H), 2.66-2.75 (m, 1 H), 3.16-3.23 (m, 3 H), 3.51 (s, 3 H, -COOC**H$_3$**), 3.53 (s, 3 H, -COOC**H$_3$**), 3.57-3.61 (m, 1 H), 3.65 (s, 3 H, -COOC**H$_3$**), 3.70 (s, 1 H),

3.76-3.82 (m, 4 H), 3.94-3.98 (m, 1 H), 4.03-4.08 (m, 1 H), 4.11-4.25 (m, 6 H), 4.50-4.55 (m, 1 H), 4.68-4.73 (m, 1 H), 4.76-4.80 (m, 3 H), 4.85-4.89 (m, 2 H), 4.96-5.02 (m, 1 H), 5.08 (s, 1 H), 5.10 (s, 2 H, Ph-C**H**-), 5.13-5.16 (m, 1 H), 5.20-5.29 (m, 5 H), 5.32-5.40 (m, 2 H), 5.42-5.46 (m, 2 H), 5.54 (s, 1 H, Ph-C**H**-), 5.65-5.79 (m, 1 H, -C**H**=CH_2), 7.34-7.40 (m, 15 H, $\mathbf{H_{ar}}$), 7.43-7.61 (m, 13 H, $\mathbf{H_{ar}}$), 7.77-8.09 (m, 17 H, $\mathbf{H_{ar}}$).

^{13}C NMR: (100 MHz, $CDCl_3$) δ [ppm] −5.1 (-Si-**C**H_3), −4.4 (-Si-**C**H_3), 17.8 (-Si-**C**$(CH_3)_3$), 22.9 (-CO-**C**H_3), 23.0 (2 C, -CO-**C**H_3), 25.5 (3 C, -Si-C(**C**$H_3)_3$), 52.2, 52.3 (-O**C**H_3), 52.9 (-O**C**H_3), 53.0 (-O**C**H_3), 58.3, 58.7, 62.9, 65.6, 67.9, 68.7, 68.8, 68.9, 70.9, 71.7, 72.6, 72.8, 73.0, 74.4, 75.0, 75.3, 76.3, 76.4, 76.7, 76.9, 77.0, 77.4, 77.5, 80.2, 81.7, 97.2 (**C-1**), 98.4 (-O-**C**H-O-), 98.8 (-O-**C**H-O-), 100.7 (Ph-**C**H-), 100.8 (2 C, Ph-**C**H-), 101.0 (-O-**C**H-O-), 101.1 (-O-**C**H-O-), 101.5 (-O-**C**H-O-), 117.9 (-CH=**C**H_2), 126.2, 128.4, 128. 7, 128. 8, 128.9, 129.2, 129.6, 129.9, 133.3, 133.4, 133.5, 133.7, 137.3, 165.0 (**C**O_{Bz}), 165.1 (**C**O_{Bz}), 165.28 (2 C, **C**O_{Bz}), 165.32 (**C**O_{Bz}), 165.7 (**C**O_{Bz}), 167.28 (-**C**OOMe), 167.34 (-**C**OOMe), 168.4 (-**C**OOMe), 170.0 (-NH-**C**O-), 170.4 (-NH-**C**O-), 170.8 (-NH-**C**O-), some peaks are overlapping.

HR–MS: (ESI positive, $CHCl_3$/MeOH) $[M+2Na]^{2+}$ calcd for $[C_{117}H_{125}N_3O_{40}SiNa_2]^{2+}$: 1142.86965, found 1142.86893.

IR: (KBr) ν_{max}= 3443 cm^{-1}, 1734, 1654, 1646, 1637, 1452, 1376, 1315, 1273, 1178, 1093, 1072, 1029, 712.

Optical rotation: $[\alpha]_D^{23}(\deg\,cm^3\,g^{-1}dm^{-1})$ = +65.6 (*c* = 1.0, $CHCl_3$).

Melting point: 189 °C

3.2 Prop-2-en-1-yl 4-*O*-acetyl-2,3-di-*O*-benzoyl-6-methyl-D-glucopyranuronosyl-(1→3)-4,6-di-*O*-acetyl-2-(acetylamino)-2-deoxy-β-D-glucopyranosyl-(1→4)-2,3-di-*O*-benzoyl-6-methyl-β-D-glucopyranuronosyl-(1→3)-4,6-di-*O*-acetyl-2-(acetylamino)-2-deoxy-β-D-glucopyranosyl-(1→4)-2,3-di-*O*-benzoyl-6-methyl-β-D-glucopyranuronosyl-(1→3)-4,6-di-*O*-acetyl-2-(acetylamino)-2-deoxy-α-D-glucopyranoside (9)

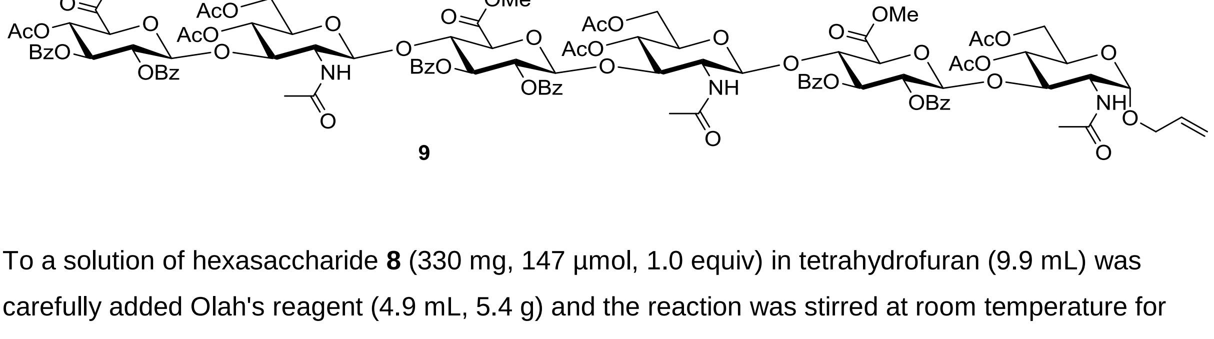

9

To a solution of hexasaccharide **8** (330 mg, 147 µmol, 1.0 equiv) in tetrahydrofuran (9.9 mL) was carefully added Olah's reagent (4.9 mL, 5.4 g) and the reaction was stirred at room temperature for

5 d. Then, the reaction mixture was diluted with dichloromethane (150 mL) and carefully quenched with saturated aqueous sodium hydrogen carbonate solution (200 mL). The organic phase was washed with saturated aqueous cupric sulfate solution (4 × 50 mL), dried over Na_2SO_4, and the solvents were removed under reduced pressure. The remaining solid was dried under high vacuum for 2 h before it was taken up in pyridine (10 mL). Then, acetic anhydride (5 mL) was added dropwise and the reaction mixture was stirred at room temperature for 15 h. The reaction mixture was diluted with dichloromethane (150 mL) and carefully quenched with ice cooled saturated aqueous sodium hydrogen carbonate solution (200 mL). Subsequently, **t**he organic phase was washed with saturated aqueous cupric sulfate solution (5 × 50 mL), dried over Na_2SO_4 and the solvents were removed under reduced pressure. The crude product was purified by column chromatography (dichloromethane/methanol, 50:1 → 10:1 v/v) and compound **9** (223 mg, 103 µmol, 70%) was obtained as white solid.

R_f: 0.25 (dichloromethane/methanol = 40:1 v/v).

^{1}H NMR: (400 MHz, $CDCl_3$) δ [ppm] 1.62-1.93 (m, 27 H, 9 × -CO-C**H_3**), 2.07-2.08 (m, 3 H, -CO-C**H_3**), 2.53-2.62 (m, 1 H), 3.33-3.38 (m, 2 H), 3.46-3.60 (m, 5 H), 3.70-3.77 (m, 9 H, 3 × -COOC**H_3**), 3.88-3.99 (m, 4 H), 4.06-4.12 (m, 3 H), 4.18-4.25 (m, 3 H), 4.53-4.69 (m, 5 H), 4.74- 4.77 (m, 1 H), 4.82-4.93 (m, 3 H), 4.98-5.03 (m, 1 H), 5.09-5.40 (m, 11 H), 5.43-5.58 (m, 3 H), 5.62-5.68 (m, 1 H), 5.77-5.91 (m, 1 H, -C**H**=CH_2), 7.30-7.35 (m, 8 H, **H_{ar}**), 7.40-7.58 (m, 10 H, **H_{ar}**), 7.80-7.91 (m, 12 H, **H_{ar}**).

^{13}C NMR: (100 MHz, $CDCl_3$) δ [ppm] 20.52 (-CO-**C**H_3), 20.56 (-CO-**C**H_3), 20.63 (-CO-**C**H_3), 20.67 (-CO-**C**H_3), 20.9 (-CO-**C**H_3), 22.8 (-CO-**C**H_3), 22.9 (-CO-**C**H_3), 23.0 (-NH-CO-**C**H_3), 23.55 (-NH-CO-**C**H_3), 23.59 (-NH-CO-**C**H_3), 52.4 (-O**C**H_3), 52.8 (-O**C**H_3), 53.0 (-O**C**H_3), 59.0, 59.1, 59.2, 62.1 (-O**C**H_2-), 62.2 (-O**C**H_2-), 62.3 (-O**C**H_2-), 65.5, 65.6, 68.3, 68.4, 68.7, 68.8, 69.3, 69.5, 69.6 (-**C**H_2-CH=CH_2), 71.9, 72.0, 72.1, 72.4, 72.6, 72.7, 73.3, 74.5, 74.7, 75.1, 75.7, 76.4, 77.7, 80.1, 80.6, 96.6, 97.5, 97.8, 100.7, 101.2, 101.3, 101.6, 118.5 (-CH=**C**H_2), 126.1, 126.2, 128.3, 128.42, 128.47, 128.53, 128.59, 128.72, 128.75, 128.84, 128.87, 129.0, 129.21, 129.23, 129.4, 129.8, 129.9, 133.3, 133.46, 133.52, 133.6, 133.8, 164.6 (**C**O_{Bz}), 164.7 (**C**O_{Bz}), 164.9 (**C**O_{Bz}), 165.3 (**C**O_{Bz}), 165.5 (**C**O_{Bz}), 165.7 (**C**O_{Bz}), 167.1 (-**C**OOMe), 167.45 (-**C**OOMe), 167.54 (-**C**OOMe), 169.29 (**C**O_{Ac}), 169.38 (**C**O_{Ac}), 169.42 (**C**O_{Ac}), 169.48 (**C**O_{Ac}), 169.6 (**C**O_{Ac}), 169.7 (**C**O_{Ac}), 170.57 (**C**O_{Ac}), 170.63 (-NH-**C**O-), 170.68 (-NH-**C**O-), 170.9 (-NH-**C**O-), some peaks are overlapping.

HR–MS: (ESI positive, $CHCl_3$/MeOH) $[M+2Na]^{2+}$ calcd for $[C_{104}H_{113}N_3O_{47}Na_2]^{2+}$: 1100.81644, found 1100.81606, $[M+3Na]^{3+}$ calcd for $[C_{104}H_{113}N_3O_{47}Na_3]^{3+}$: 741.54070, found 741.54175.

IR: (KBr) ν_{max}= 3444 cm^{-1}, 1736, 1654, 1452, 1373, 1275, 1109, 1094, 1070, 1042, 1030, 714.

Optical rotation: $[\alpha]_D^{23}(\deg\,cm^3\,g^{-1}dm^{-1})$ = +29.2 (c = 1.0, $CHCl_3$).

Melting point: 178 °C

3.3 3-Azido-2-hydroxypropyl 4-*O*-acetyl-2,3-di-*O*-benzoyl-6-methyl-D-glucopyranuronosyl-(1→3)-4,6-di-*O*-acetyl-2-(acetylamino)-2-deoxy-β-D-glucopyranosyl-(1→4)-2,3-di-*O*-benzoyl-6-methyl-β-D-glucopyranuronosyl-(1→3)-4,6-di-*O*-acetyl-2-(acetylamino)-2-deoxy-β-D-glucopyranosyl-(1→4)-2,3-di-*O*-benzoyl-6-methyl-β-D-glucopyranuronosyl-(1→3)-4,6-di-*O*-acetyl-2-(acetylamino)-2-deoxy-α-D-glucopyranoside (10)

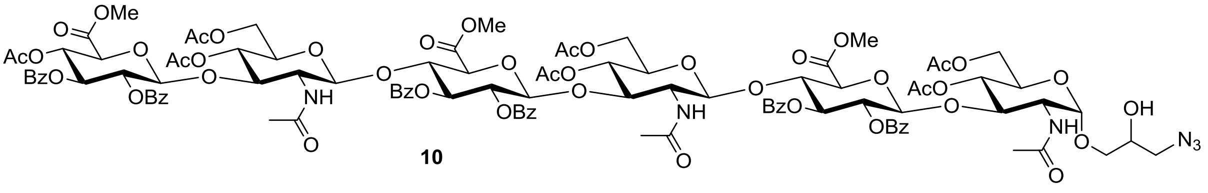

Hexasaccharide **9** (222 mg, 102 µmol, 1.0 equiv) was dissolved in acetone (2.0 mL) and the solution was cooled to −78 °C. Murray's reagent (0.1 M solution in acetone; 3.1 mL, 310 µmol, 3.0 equiv) was added and the reaction mixture was left to slowly warm to room temperature and was stirred for 22 h. An additional amount of Murray's reagent (0.1 M solution in acetone; 3.1 mL, 310 µmol, 3.0 equiv) was added and the reaction mixture was stirred for additional 2 d. All volatiles were removed under reduced pressure and the solid residue was dried under high vacuum for 6 h. Then, it was dissolved in *N*,*N*-dimethylformamide (2.3 mL) and sodium azide (19.9 mg, 306 mmol, 3.0 equiv) was added before the reaction mixture was stirred at room temperature for 2 d. Afterwards, it was diluted with dichloromethane (20 mL) and washed with saturated aqueous sodium hydrogen carbonate solution (20 mL), brine (20 mL) and water (2 × 15 mL). The organic phase was dried over Na_2SO_4 and the solvents were removed under reduced pressure. The crude product was purified by column chromatography (dichloromethane/methanol, 50:1 → 20:1 v/v) to give final product **10** (158 mg, 71.4 µmol, 70%) as white solid.

R_f: 0.30 (dichloromethane/methanol = 20:1 *v/v*).

^{1}H NMR: (400 MHz, $CDCl_3$) δ [ppm] 1.64-2.07 (m, 30 H, 10 × -CO-C**H₃**), 2.52-2.73 (m, 2 H), 3.31 (s, 1 H, -C**H₂**-N₃), 3.36-3.62 (m, 9 H), 3.66-3.75 (m, 9 H, 3 × -COOC**H₃**), 3.86-4.30 (m, 14 H), 4.55-4.72 (m, 5 H), 4.77-5.03 (m, 6 H), 5.15-5.54 (m, 8 H), 7.30-7.53 (m, 18 H, **H$_{ar}$**), 7.81-7.94 (m, 12 H, **H$_{ar}$**).

^{13}C NMR: (100 MHz, $CDCl_3$) δ [ppm] 20.6 (-CO-**C**H_3), 20.66 (-CO-**C**H_3), 20.69 (-CO-**C**H_3), 20.8 (-CO-**C**H_3), 20.94 (-CO-**C**H_3), 20.98 (2 × -CO-**C**H_3), 23.0 (-NH-CO-**C**H_3), 23.5 (-NH-CO-**C**H_3), 23.7 (-NH-CO-**C**H_3), 52.8 (-O**C**H_3), 52.96 (-O**C**H_3), 53.03 (-O**C**H_3), 53.5 (-**C**H_2-N_3), 58.9, 59.1, 59.2, 62.1 (-O**C**H_2-), 62.2 (-O**C**H_2-), 62.4 (-O**C**H_2-), 68.3, 68.5, 68.7, 68.9, 69.0, 69.3, 69.4, 69.5, 69.7, 70.2, 70.34, 70.36, 70.5, 71.9, 72.2, 72.5, 72.7, 72.9, 74.8, 75.2, 75.7, 75.9, 97.5, 97.8, 98.1, 100.6, 101.0, 101.5, 126.0, 128.4, 128.5, 128.6, 128.7, 128.8, 129.1, 129.3, 129.7, 129.8, 133.5, 133.6, 133.8, 164.6 (**C**O$_{Bz}$), 164.7 (**C**O$_{Bz}$), 165.4 (3 C, **C**O$_{Bz}$), 165.6 (**C**O$_{Bz}$), 167.0 (-**C**OOMe), 167.4 (-**C**OOMe), 167.6

(-**C**OOMe), 169.49 (2 × **C**O_{Ac}), 169.54 (**C**O_{Ac}), 169.8 (**C**O_{Ac}), 170.7 (**C**O_{Ac}), 170.78 (**C**O_{Ac}), 170.80 (**C**O_{Ac}), 171.1 (-NH-**C**O-), 171.68 (-NH-**C**O-), 171.73 (-NH-**C**O-), some peaks are overlapping.

HR–MS: (ESI positive, $CHCl_3$/MeOH) $[M+2H]^{2+}$ calcd for $[C_{104}H_{116}N_6O_{48}]^{2+}$: 1108.34048, found 1108.34056, $[M+H+Na]^{2+}$ calcd for $[C_{104}H_{115}N_6O_{48}Na]^{2+}$: 1119.33145, found 1119.33170, $[M+2Na]^{2+}$ calcd for $[C_{104}H_{114}N_6O_{48}Na_2]^{2+}$: 1130.32242, found 1130.32270.

IR: (KBr) ν_{max}= 3443 cm^{-1}, 2105, 1736, 1655, 1452, 1373, 1274, 1110, 1095, 1070, 1045, 714.

Melting point: 176 °C

4. NMR spectra

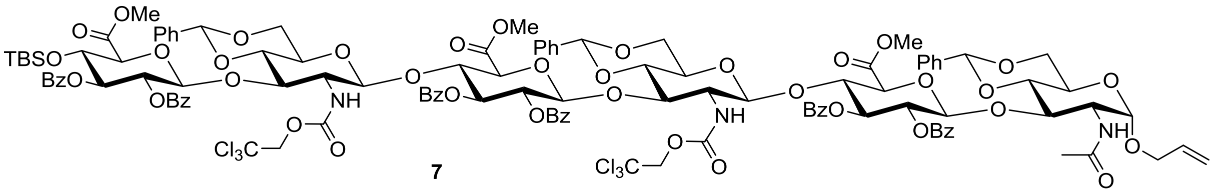

7

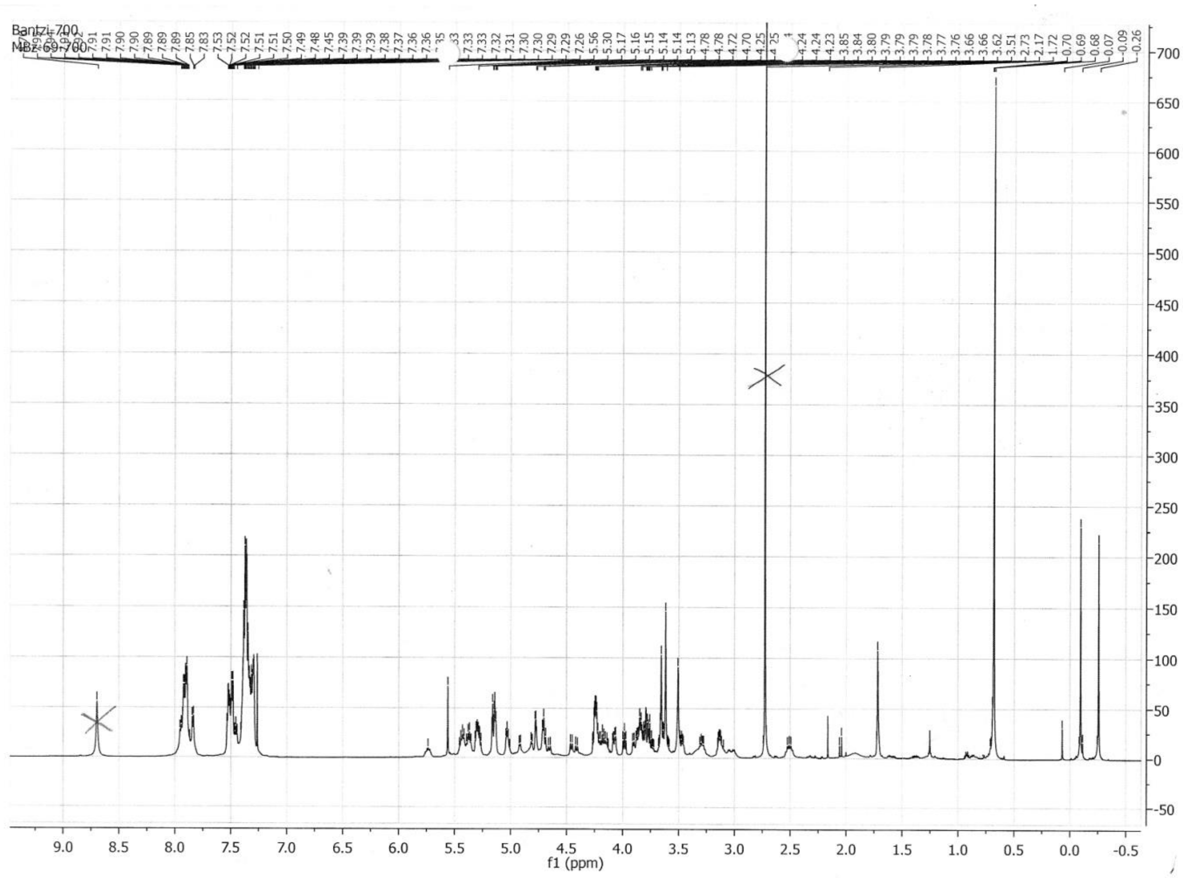

<u>Notice</u>: Peaks 2.73 and 8.70 belong to succinimide (NMR spectra of the crude intermediate **7**).

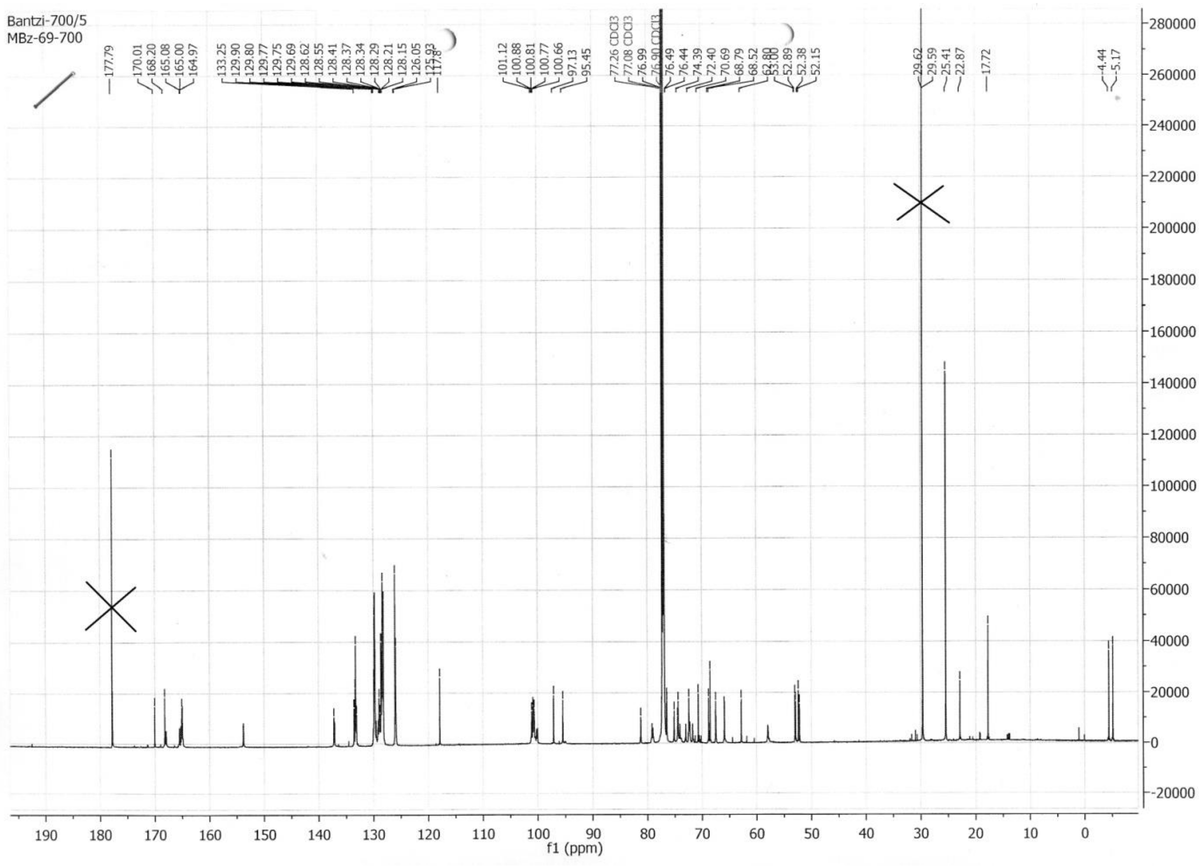

<u>Note</u>: Peaks 29.6 and 177.8 belong to succinimide (NMR spectra of the crude intermediate **7**).

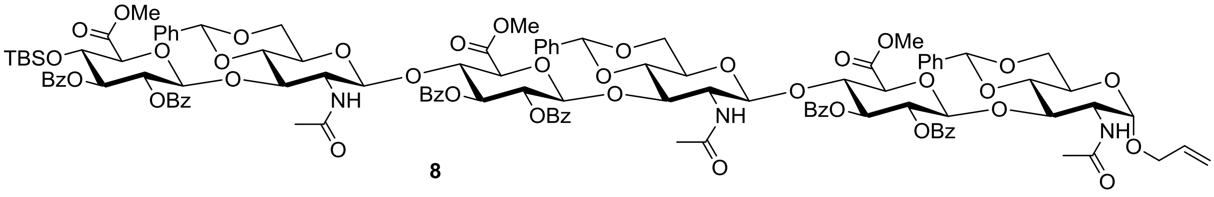
OMe
Ph
O
TBSO
BzO
OBz
NH
O
BzO
OBz
NH
O
BzO
OBz
NH
O

8

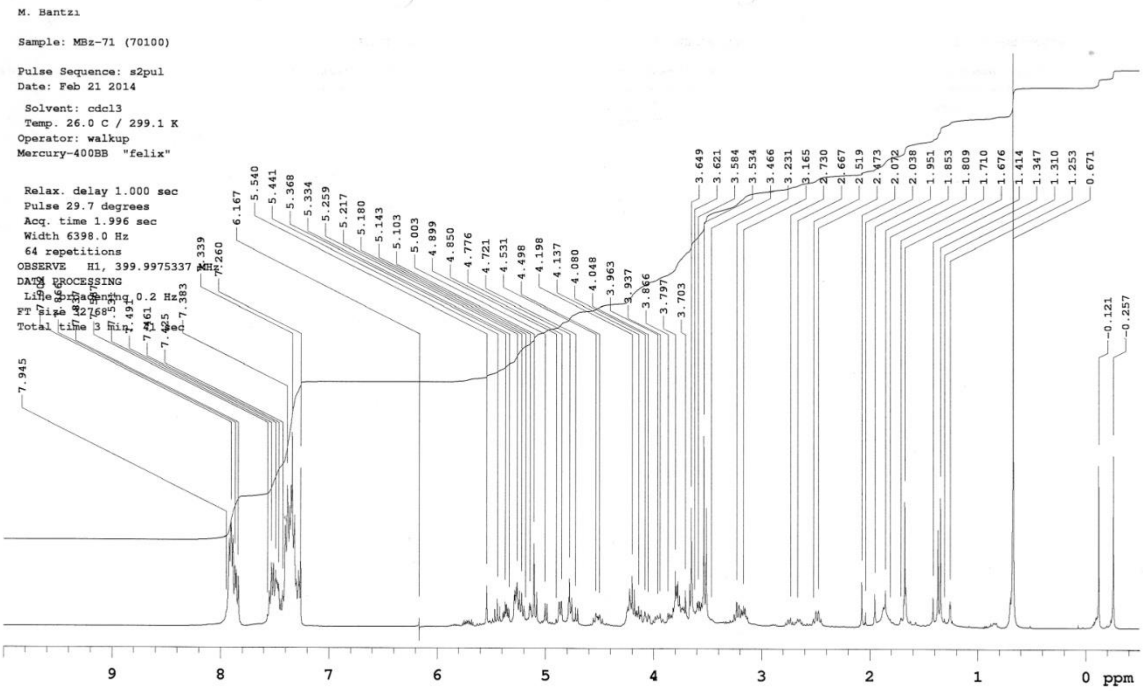
M. Bantzi
Sample: MBz-71 (70100)
Pulse Sequence: s2pul
Date: Feb 21 2014
Solvent: cdcl3
Temp. 26.0 C / 299.1 K
Operator: walkup
Mercury-400BB "felix"
Relax. delay 1.000 sec
Pulse 29.7 degrees
Acq. time 1.996 sec
Width 6398.0 Hz
64 repetitions
OBSERVE H1, 399.9975337 MHz
DATA PROCESSING
Line broadening 0.2 Hz
FT size 32768
Total time 3 min, 1 sec
7.945
7.491
7.461
7.425
7.383
7.339
7.260
6.167
5.540
5.441
5.368
5.334
5.259
5.217
5.180
5.143
5.103
5.003
4.899
4.850
4.776
4.721
4.531
4.498
4.198
4.137
4.080
4.048
3.963
3.937
3.866
3.797
3.703
3.649
3.621
3.584
3.534
3.466
3.231
3.165
2.730
2.667
2.519
2.473
2.072
2.038
1.951
1.853
1.809
1.710
1.676
1.414
1.347
1.310
1.253
0.671
-0.121
-0.257
9
8
7
6
5
4
3
2
1
0 ppm

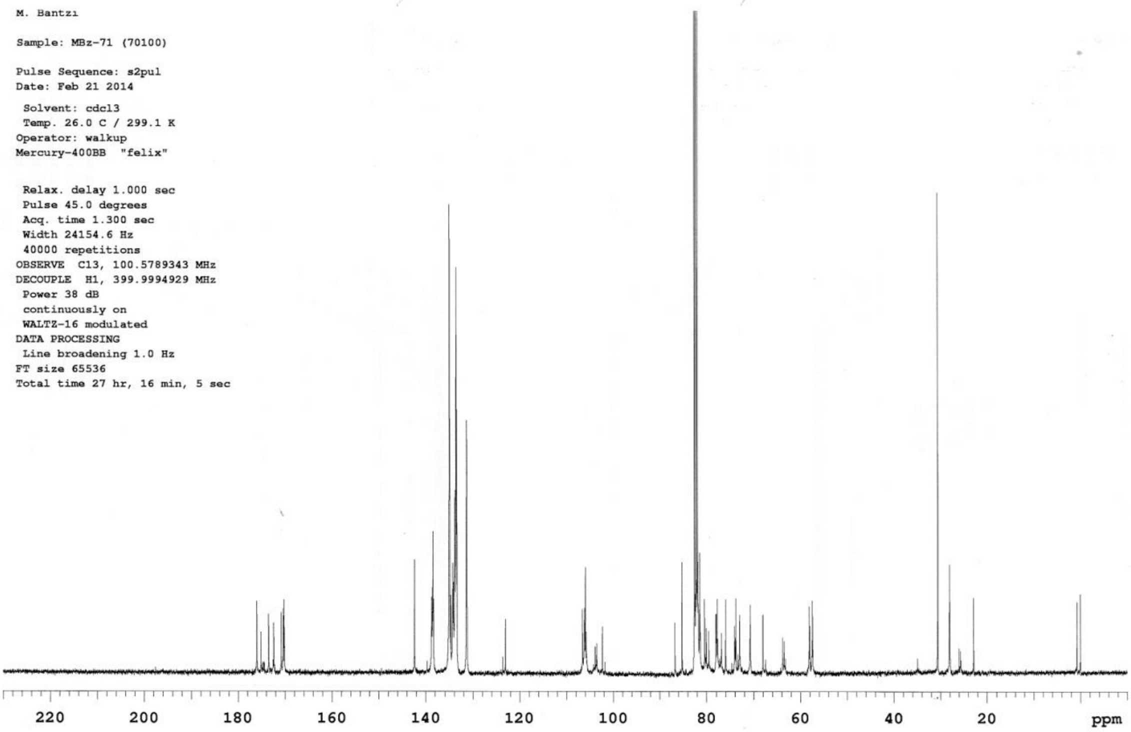
M. Bantzi
Sample: MBz-71 (70100)
Pulse Sequence: s2pul
Date: Feb 21 2014
Solvent: cdcl3
Temp. 26.0 C / 299.1 K
Operator: walkup
Mercury-400BB "felix"
Relax. delay 1.000 sec
Pulse 45.0 degrees
Acq. time 1.300 sec
Width 24154.6 Hz
40000 repetitions
OBSERVE C13, 100.5789343 MHz
DECOUPLE H1, 399.9994929 MHz
Power 38 dB
continuously on
WALTZ-16 modulated
DATA PROCESSING
Line broadening 1.0 Hz
FT size 65536
Total time 27 hr, 16 min, 5 sec
220
200
180
160
140
120
100
80
60
40
20
ppm

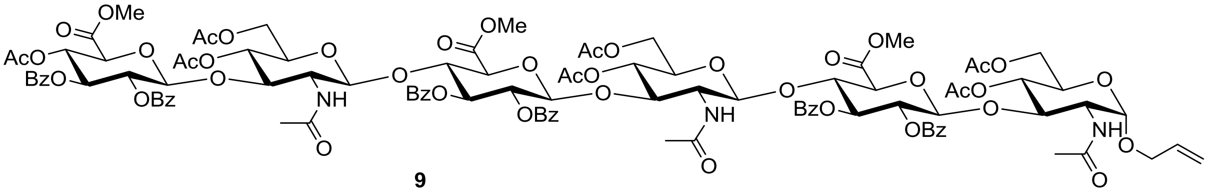

9

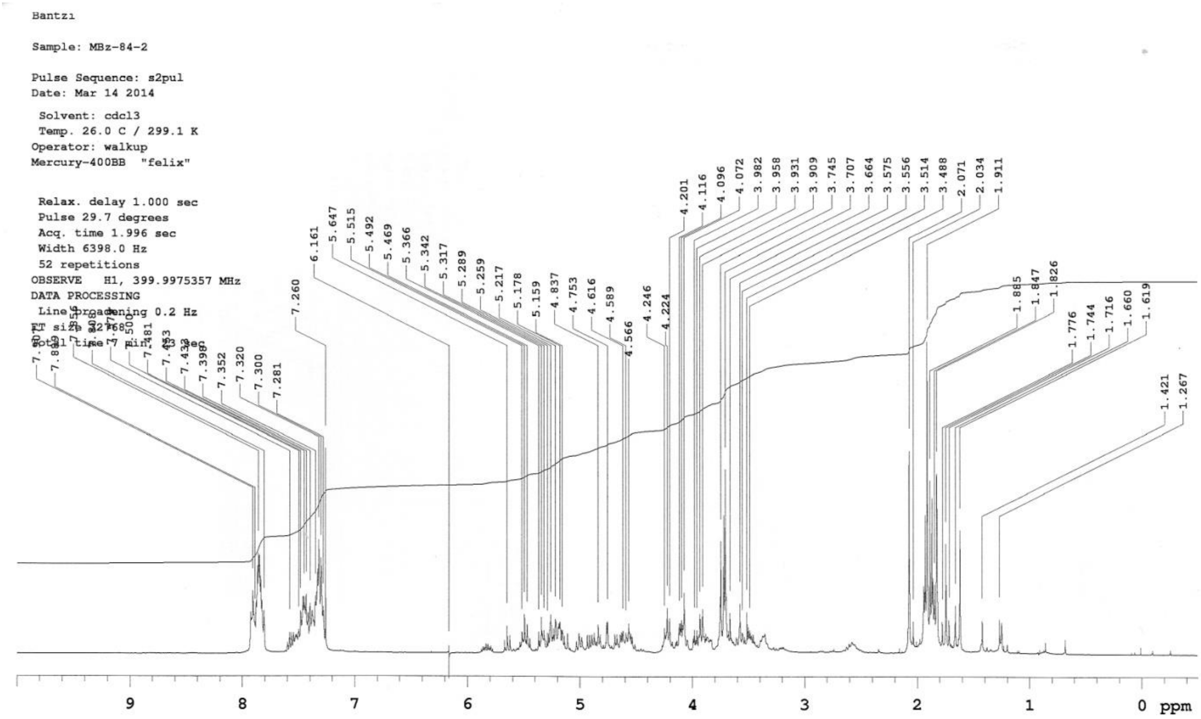

Bantzi
Sample: MBz-84-2
Pulse Sequence: s2pul
Date: Mar 14 2014
Solvent: cdcl3
Temp. 26.0 C / 299.1 K
Operator: walkup
Mercury-400BB "felix"
Relax. delay 1.000 sec
Pulse 29.7 degrees
Acq. time 1.996 sec
Width 6398.0 Hz
52 repetitions
OBSERVE H1, 399.9975357 MHz
DATA PROCESSING
Line broadening 0.2 Hz
7.352
7.320
7.300
7.281
7.260
6.161
5.647
5.515
5.492
5.469
5.366
5.342
5.317
5.289
5.259
5.217
5.178
5.159
4.837
4.753
4.616
4.589
4.566
4.246
4.224
4.201
4.116
4.096
4.072
3.982
3.958
3.931
3.909
3.745
3.707
3.664
3.575
3.556
3.514
3.488
2.071
2.034
1.911
1.885
1.847
1.826
1.776
1.744
1.716
1.660
1.619
1.421
1.267
9
8
7
6
5
4
3
2
1
0 ppm

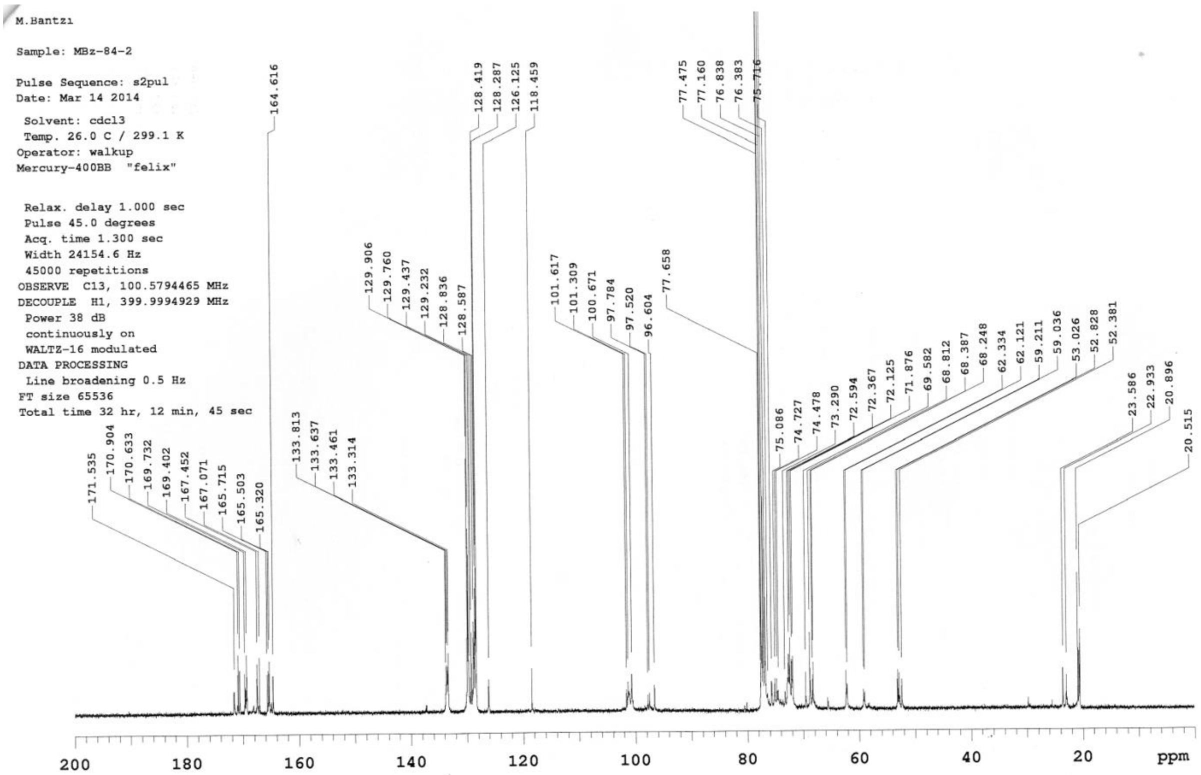

M.Bantzi
Sample: MBz-84-2
Pulse Sequence: s2pul
Date: Mar 14 2014
Solvent: cdcl3
Temp. 26.0 C / 299.1 K
Operator: walkup
Mercury-400BB "felix"
Relax. delay 1.000 sec
Pulse 45.0 degrees
Acq. time 1.300 sec
Width 24154.6 Hz
45000 repetitions
OBSERVE C13, 100.5794465 MHz
DECOUPLE H1, 399.9994929 MHz
Power 38 dB
continuously on
WALTZ-16 modulated
DATA PROCESSING
Line broadening 0.5 Hz
FT size 65536
Total time 32 hr, 12 min, 45 sec
171.535
170.904
170.633
169.732
169.402
167.452
167.071
165.715
165.503
165.320
164.616
133.813
133.637
133.461
133.314
129.906
129.760
129.437
129.232
128.836
128.587
128.419
128.287
126.125
118.459
101.617
101.309
100.671
97.784
97.520
96.604
77.658
77.475
77.160
76.838
76.383
75.716
75.086
74.727
74.478
73.290
72.594
72.367
72.125
71.876
69.582
68.812
68.387
68.248
62.334
62.121
59.211
59.036
53.026
52.828
52.381
23.586
22.933
20.896
20.515
200
180
160
140
120
100
80
60
40
20
ppm

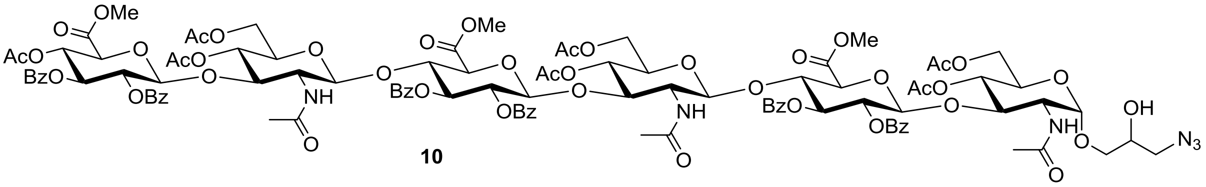
OMe
AcO
BzO
OBz
NH
O
N3
OH

10

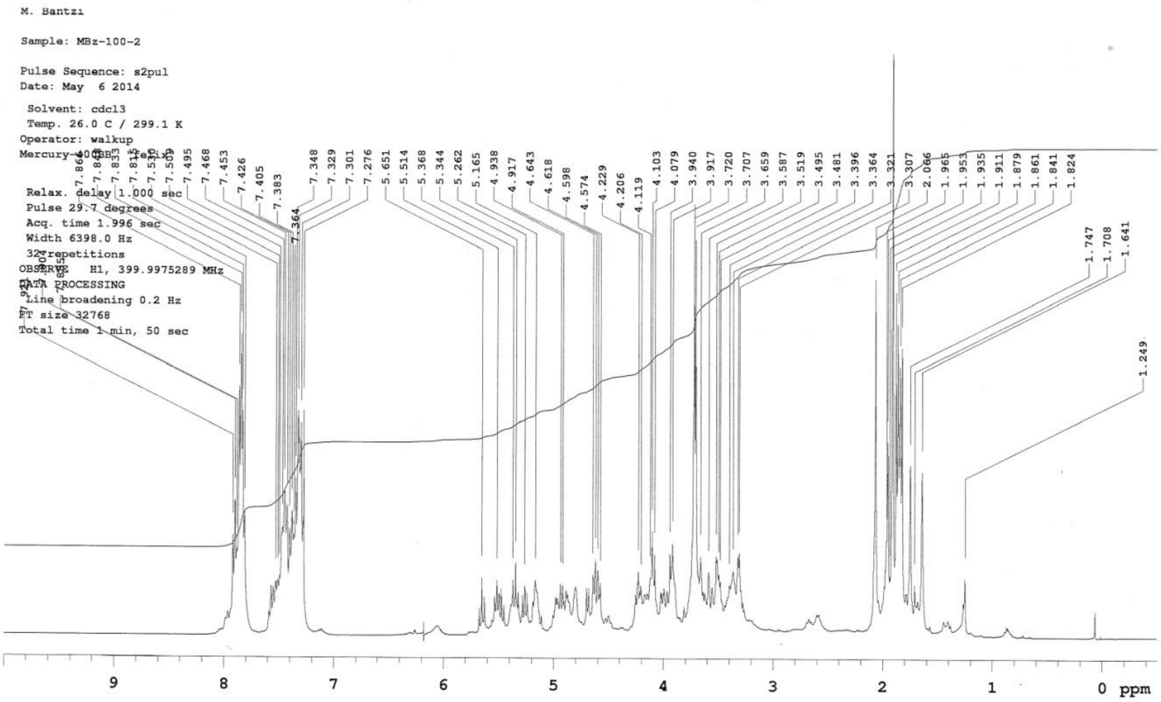
M. Bantzi
Sample: MBz-100-2
Pulse Sequence: s2pul
Date: May 6 2014
Solvent: cdcl3
Temp. 26.0 C / 299.1 K
Operator: walkup
Mercury-400BB "felix"
Relax. delay 1.000 sec
Pulse 29.7 degrees
Acq. time 1.996 sec
Width 6398.0 Hz
32 repetitions
OBSERVE H1, 399.9975289 MHz
DATA PROCESSING
Line broadening 0.2 Hz
FT size 32768
Total time 1 min, 50 sec
7.495 7.468 7.453 7.426 7.405 7.383 7.364 7.348 7.329 7.301 7.276 5.651 5.514 5.368 5.344 5.262 5.165 4.938 4.917 4.643 4.618 4.598 4.574 4.229 4.206 4.119 4.103 4.079 3.940 3.917 3.720 3.707 3.659 3.587 3.519 3.495 3.481 3.396 3.364 3.321 3.307 2.066 1.965 1.953 1.935 1.911 1.879 1.861 1.841 1.824
1.747 1.708 1.641
1.249
9 8 7 6 5 4 3 2 1 0 ppm

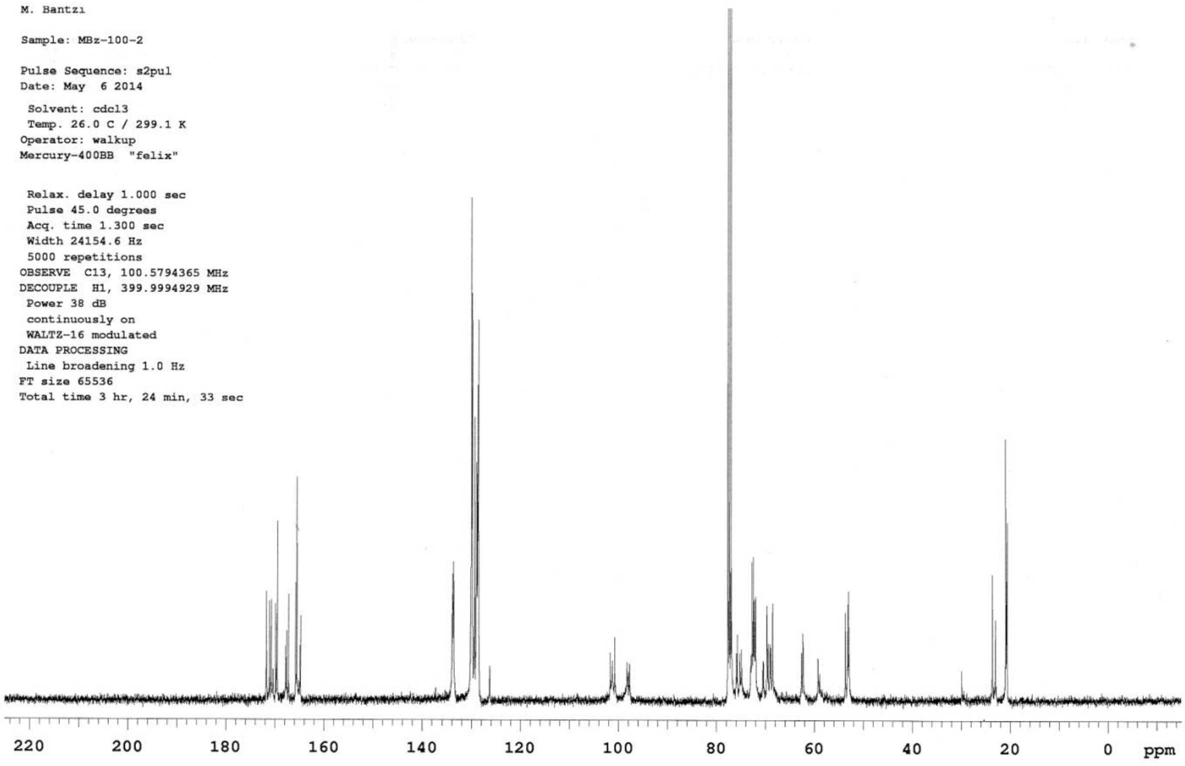
M. Bantzi
Sample: MBz-100-2
Pulse Sequence: s2pul
Date: May 6 2014
Solvent: cdcl3
Temp. 26.0 C / 299.1 K
Operator: walkup
Mercury-400BB "felix"
Relax. delay 1.000 sec
Pulse 45.0 degrees
Acq. time 1.300 sec
Width 24154.6 Hz
5000 repetitions
OBSERVE C13, 100.5794365 MHz
DECOUPLE H1, 399.9994929 MHz
Power 38 dB
continuously on
WALTZ-16 modulated
DATA PROCESSING
Line broadening 1.0 Hz
FT size 65536
Total time 3 hr, 24 min, 33 sec
220 200 180 160 140 120 100 80 60 40 20 0 ppm

5. References

1. France, R. R.; Compton, R. G.; Davis, B. G.; Fairbanks, A. J.; Rees, N. V.; Wadhawan, J. D. *Org. Biomol. Chem.,* **2004,** *2,* 2195-2202.

2. Tully, S. E.; Mabon, R.; Gama, C. I.; Tsai, S. M.; Liu, X. W.; Hsieh-Wilson, L. C. *J. Am. Chem. Soc.,* **2004,** *126,* 7736-7737.

3. Wang, Z.; Zhou, L. Y.; El-Boubbou, K.; Ye, X. S.; Huang, X. F. *J. Org. Chem.,* **2007,** *72,* 6409-6420.

4. Geng, Y. Q.; Zhang, L. H.; Ye, X. S. *Tetrahedron,* **2008,** *64,* 4949-4958.

5. Izumi, M.; Fukase, K.; Kusumoto, S., *Biosci. Biotechnol. Biochem.* **2002,** *66,* 211-214.

6. Rigol, S.; Xia, L.; Giannis, A. *Bioorg. Med. Chem.,* **2013,** *21,* 733-741.
